# Development and evaluation of a microcapsule-based phantom simulating intra- and extracellular water diffusion

**DOI:** 10.1186/s41747-026-00849-1

**Published:** 2026-11-13

**Authors:** Kazuya Sakoda, Takashi Iwanaga, Hirokazu Otsuka, Masahiko Toyota, Kiyohisa Kamimura, Koji Takumi, Fumitaka Ejima, Tsubasa Nakano, Hiroshi Imai, Thorsten Feiweier, Takashi Yoshiura

**Affiliations:** 1 Department of Radiology, Kagoshima University Graduate School of Medical and Dental Sciences, Kagoshima, Japan; 2 Department of Radiological Technology, Kagoshima Medical Technology College, Kagoshima, Japan; 3 Department of Radiological Sciences, School of Health Science, Fukushima Medical University, Fukushima, Japan; 4 Department of Radiological Technology, Kagoshima University Hospital, Kagoshima, Japan; 5 Department of Advanced Radiological Imaging, Kagoshima University Graduate School of Medical and Dental Sciences, Kagoshima, Japan; 6 Research & Collaboration, Siemens Healthcare K.K., Tokyo, Japan; 7 Research & Clinical Translation, Siemens Healthineers, Erlangen, Germany

**Keywords:** Diffusion magnetic resonance imaging, IMPULSED, Microstructure, Oscillating gradient spin-echo, Pulsed gradient spin-echo

## Abstract

**Objectives:**

To develop a microcapsule-based phantom that simulates intra- and extracellular diffusion and to evaluate its microstructural validity using time-dependent diffusion-weighted imaging (DWI) metrics and limited spectrally edited diffusion (IMPULSED) modeling.

**Materials and methods:**

Microcapsules with undecane cores and melamine resin membranes were fabricated in three diameter ranges and dispersed at different concentrations within an octane gel to create phantoms with controlled microstructure. DWI was performed using pulsed gradient spin-echo and oscillating gradient spin-echo research sequences with effective diffusion times of 5.8, 10.2, and 51.1 ms. Apparent diffusion coefficient (ADC), relative ADC (rADC), and incremental area under the curve (iAUC) were calculated. Microstructural parameters, including diameter, intracellular volume fraction (*f*_in_), intracellular diffusivity (*D*_in_), extracellular diffusivity (*D*_ex_), and cellularity were estimated using the IMPULSED model. Group differences were assessed using one-way ANOVA with Tukey’s post hoc test. Pearson’s correlation analysis was conducted to evaluate correlations between theoretical and estimated microstructural parameters. Reproducibility was assessed with Bland–Altman analysis according to repeated imaging of independently fabricated phantoms.

**Results:**

ADC significantly decreased with increasing diffusion time in all microcapsule phantoms (*p* < 0.001), whereas reference materials showed no time dependence. Higher microcapsule density resulted in lower ADC and higher rADC and iAUC. IMPULSED-derived diameters closely matched theoretical values (*r* = 0.992). Estimated *f*_in_ and cellularity strongly correlated with theoretical values (*r* = 0.940 and 0.910). Reproducibility was good for ADC and IMPULSED-derived parameters.

**Conclusion:**

This microcapsule-based phantom reproduces diffusion time-dependent restriction and enables quantitative validation of time-dependent DWI and IMPULSED-derived microstructural metrics.

**Key Points:**

***Question*** Validation of time-dependent diffusion MRI and microstructural models requires phantoms that reproduce both intra- and extracellular water diffusion under controlled conditions.

***Findings*** The microcapsule-based phantom reproduced diffusion-time-dependent restriction, and MRI-estimated diameters strongly correlated with theoretical diameters across tested conditions, with a Pearson correlation coefficient of 0.992.

***Relevance Statement*** The microcapsule-based phantom provides controlled intra- and extracellular diffusion environments for technical validation and standardization of quantitative MRI methods designed to characterize tissue microstructure.

**Graphical Abstract:**

**Figure:**
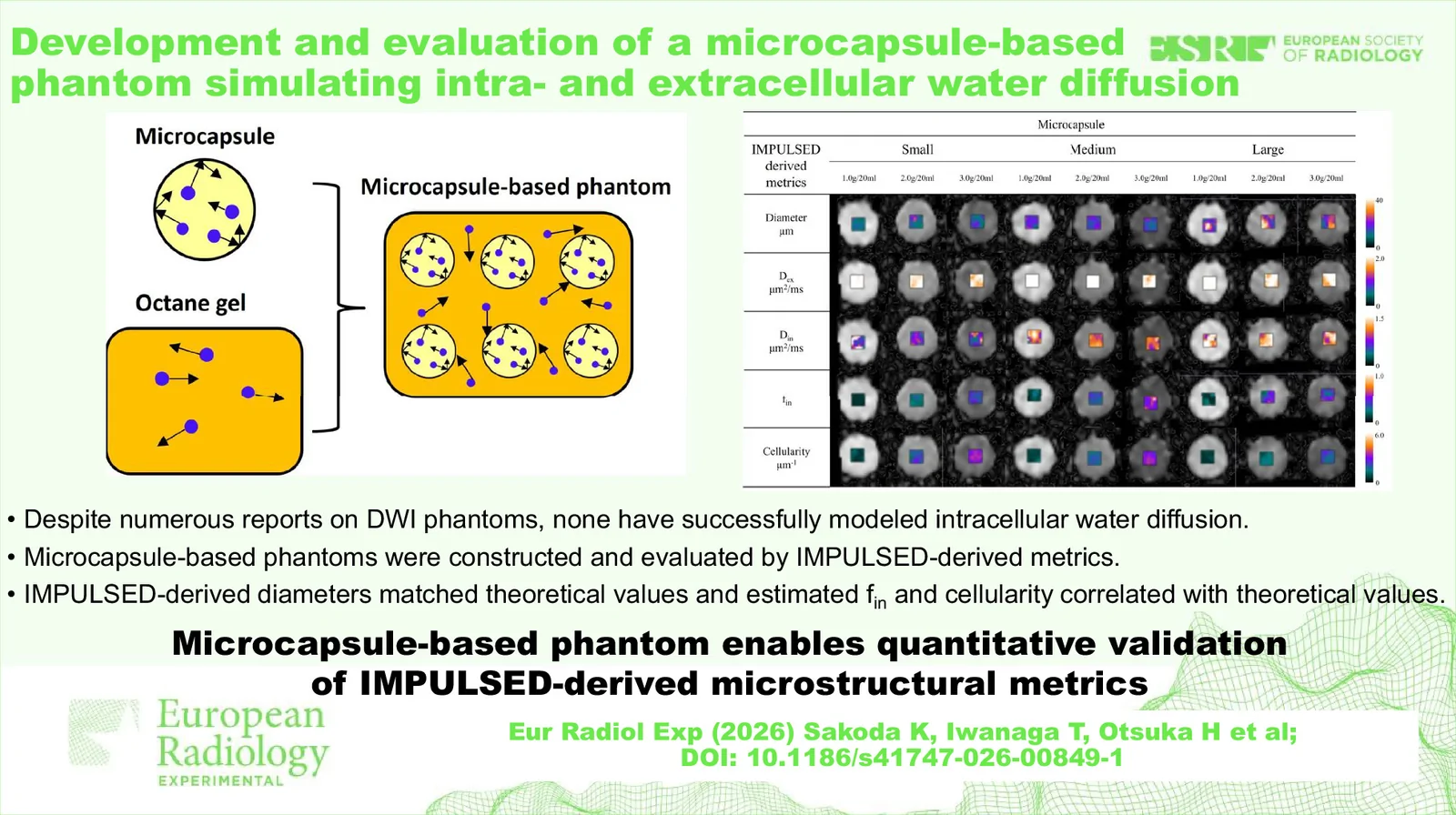

## Introduction

Diffusion-weighted imaging (DWI) enables water diffusion characterization and provides insight into tissue microstructure and pathology [1, 2]. The apparent diffusion coefficient (ADC) is used for tumor characterization and prognostic assessment [3, 4]. The ADC measured with DWI relies on effective diffusion time, which is the duration allowed for water molecules to probe their microenvironment [5]. Conventional DWI is performed using pulsed gradient spin echo (PGSE) with a diffusion time of 20–40 ms [6]. At these relatively long timescales, water molecules interact with multiple cellular boundaries, making the ADC sensitive to the overall density of the tissue but less specific to individual cell sizes. Oscillating gradient spin-echo (OGSE) sequences can shorten diffusion times by replacing the long-lasting diffusion-sensitizing gradients used in PGSE methods with rapidly oscillating gradients [7–9]. Hence, OGSE captures water motion before it is heavily restricted by cell membranes, probing water diffusion over a shorter length scale than PGSE [10]. Time-dependent MRI using PGSE and OGSE provides microstructural information beyond conventional DWI [11–15]. The imaging microstructural parameters using the limited spectrally edited diffusion (IMPULSED) model exploit the diffusion-time dependence of ADC obtained from PGSE and OGSE acquisition [16, 17]. IMPULSED is a two-compartment model that assumes the DWI signal originates solely from intra- and extracellular compartments without water exchange through the membrane. This model characterizes microstructural features utilizing quantitative metrics, including cell diameter, cellularity, intracellular volume fraction (*f*_in_), intracellular intrinsic diffusivity (*D*_in_), and apparent extracellular diffusivity (*D*_ex_), which have demonstrated utility as non-invasive imaging biomarkers associated with histopathology [17–23].

A phantom with diffusion characteristics that simulate human tissues can be of great value in advancing DWI research. Previously proposed DWI phantoms have used materials, including sucrose [24], polyvinylpyrrolidone [25], and alkanes [26], in which ADC is primarily governed by solution viscosity and temperature. Conversely, phantoms composed of glass or polyethylene beads [27, 28] possess physical barriers; however, the beads themselves produce no MR signal. Thus, existing phantoms simulate extracellular hindered water diffusion. To date, the proposed DWI phantoms have not incorporated intracellular water diffusion. Microcapsules encapsulate water-insoluble substances within their internal core [29]. They are widely used across the pharmaceutical, cosmetic, food, and chemical industries as an encapsulation technology that enables the protection and controlled release of liquid or solid core substances [29]. Microcapsules contain an MRI signal source inside and have an impermeable membrane. Therefore, we hypothesized that microcapsule-based phantoms simulate both intra- and extracellular diffusion restriction. The current study aimed to develop a phantom that simulates intra- and extracellular diffusion restriction using microcapsules and evaluate it using time-dependent DWI- and IMPULSED model analysis-derived metrics.

## Materials and methods

### Phantom

**Fig. 1:**
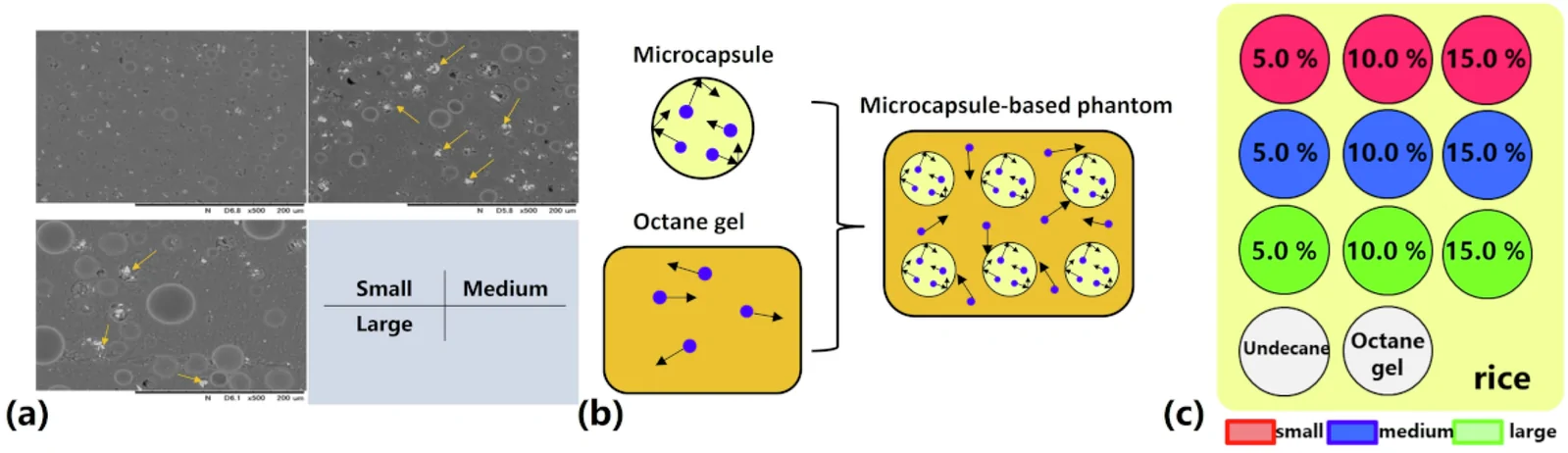
**a** Microscopic images of the microcapsules utilized in this study. Three size groups were defined (“small,” “medium,” and “large”), with corresponding mean diameters of 10.50, 21.56, and 31.00 μm, respectively. Residual melamine shell material was observed in all images (arrows). **b** Schematic illustration of the proposed phantom. Intracellular diffusion is replicated by microcapsules comprising undecane, whereas extracellular diffusion is simulated using an octane gel. **c** Overview of the phantom. Each type of microcapsule was dispersed in the octane gel at different concentrations. Octane gel and undecane were also prepared as reference materials

Microcapsules, comprising an undecane core encapsulated by a melamine resin membrane, were custom-manufactured by Japan Capsular Products, Inc. (https://www.japancapsular.co.jp/) via emulsion-based *in situ* polymerization. Undecane was selected as the intracapsular material because its ADC at MR scanner room temperature is approximately 1.0 μm²/ms [26], which closely matches the *D*_in_ typically assumed in the IMPULSED model [18]. Microcapsules were categorized into three diameter groups, representing clinically relevant tumor cell sizes [30, 31], with mean outer diameters of 10.50, 21.56, and 31.00 μm (“small,” “medium,” and “large”) (Fig. 1a). The membrane thickness was engineered to vary with the microcapsule size, as larger microcapsules require a more robust shell to maintain their integrity during the fabrication process. A laser diffraction particle size analyzer (LMS-2,000e; Seishin Enterprise Co., Ltd.) was used to measure these outer diameters. Microcapsules of varying concentrations (5.0%, 10.0%, and 15.0% (w/v)) were dispersed in an octane gel prepared with 5% palmitic acid dextrin (Rheopearl, type KL2; Chiba Flour Milling Co., Ltd.) to prevent sedimentation for each size category. To ensure a 20 mL total volume, the required mass of microcapsules (calculated based on their respective specific gravities: 0.89 for small, 0.91 for medium, and 0.93 for large) was mixed with octane gel. Octane has an ADC at MR room temperature of approximately 2.0 μm²/ms [26], which is comparable to the extracellular diffusion coefficient typically assumed in simulation studies. The samples were then stirred at 1,000 rpm for 10 min using a magnetic stirrer (RCH-1,000; EYELA Tokyo Rikakikai Co., Ltd.). To assess the reproducibility, two phantoms for each combination of three microcapsule diameters and three concentrations were independently prepared, generating 18 phantoms in total. Octane gel and undecane were prepared as reference materials. To reduce magnetic susceptibility-related geometric distortion, each phantom was surrounded by rice [32]. Figure 1b, c illustrate an overview of the phantoms utilized in this study.

### Measurement of membrane thickness of microcapsules and calculation of microcapsular volume fraction and density

Microcapsule membrane thickness and outer diameter were measured by a technician at the Kagoshima Prefectural Institute of Industrial Technology (Kagoshima, Japan) using a digital microscope (RH-2,000; HIROX Co., Ltd.) to ensure objective evaluation. These measurements were performed because the melamine resin membrane contributes negligibly to the MRI signal. Therefore, the diffusion signal was assumed to originate primarily from the internal undecane core. To estimate the internal diameter of the microcapsules (the effective signal source size) from the measured outer diameter, representative data on membrane thickness for several size categories were obtained to provide reference values for the internal dimensions of the signal source. Internal diameters were calculated by subtracting the membrane thickness from the measured outer diameters and utilized to estimate the microcapsule volume fraction and density within the phantom. The volume fraction was defined as follows:

$${{\mbox{Volume}}}\, {{{\rm{fraction}}}}=\frac{4}{3}\pi \times {\left({{{\rm{internal}}}}\,{{\mbox{diameter}}}\right)}^{3}\times f/{phantom}\,{total}\,{volume}$$

where f represents the distribution ratio for a given microcapsule diameter (Supplemental Digital Content 1 [CSV data]), and phantom total volume refers to the phantom volume, excluding the volume occupied by the microcapsule membrane thickness. The microcapsule density was calculated as follows:

$${\mbox{Density}}=\frac{{\mbox{Volume\, fraction}}}{{{\mathrm{internal}}}\,{\mbox{diameter}}}\,\times \,100$$

These values were considered the theoretical volume fraction and density of microcapsules within the phantom.

### MR acquisition

Phantoms were equilibrated to scanner room temperature for 24 h before imaging. After placing the phantoms in the center of the static magnetic field for 20 min, scanning was started. DWI acquisitions were repeated five times for each diffusion time. Temperature was monitored every 2 min using an optical fiber thermometer (Anritsu Meter Co., Ltd., Kanagawa, Japan).

A 3-T MR scanner (MAGNETOM Cima.X; Siemens Healthineers AG; maximum gradient amplitude = 200 mT/m, maximum slew rate = 200 T/m/s for each gradient axis) with a 20-channel head-neck coil was used in this study. All imaging was performed in coronal slices. A single-shot echo-planar imaging research sequence was used to implement both PGSE and OGSE DWI (Supplemental Digital Content 2 [Figure]). OGSE diffusion encoding used trapezoid-cosine waveforms with oscillating frequencies of 40 Hz (effective diffusion time [Δ_eff_] = 5.8 ms) and 20 Hz (Δ_eff_ = 10.2 ms), whereas the Δ_eff_ for PGSE was 51.1 ms. The PGSE and OGSE sequences shared the following imaging parameters: repetition time (TR) = 3,000 ms; echo time (TE) = 144 ms; field of view (FOV) = 160 × 53 mm^2^; reduced FOV technique (ZOOMit); voxel size = 1.8 × 1.8 × 5.0 mm^3^; *b*-value = 0, 500, 1,000, 1,500 s/mm^2^; diffusion encoding directions = 3; GRAPPA factor = 2; bandwidth = 2,604 Hz/pixel. The acquisition time was 1 min 36 s for each diffusion encoding condition (PGSE, OGSE 20 Hz, and OGSE 40 Hz), leading to a total acquisition time of 4 min 48 s. Further, a multi-echo spin-echo sequence with 16 echoes (TE, 11.7–374.4 ms, 11.7 ms echo spacing) was conducted for T_2_ value measurements with the following parameters: TR = 5,000 ms; FOV = 160 × 160 mm^2^; voxel size = 1 × 1 × 5 mm^3^; bandwidth = 228 Hz/pixel; acquisition time = 16 min 55 s.

### Image analysis

The time-dependent diffusion MRI signal was fitted employing the IMPULSED model, expressed as follows:

$$S={S}_{{in}}+{S}_{{ex}}$$

where *S*_in_ and *S*_ex_ denote the restricted and hindered signals in the intra- and extracellular compartments, respectively. The corresponding signal attenuation is calculated as follows:

$${S}_{{in}}={S}_{{in},0}={v}_{{in}}{S}_{0}\exp (-b\times {AD}{C}_{r})$$

$${S}_{{ex}}={S}_{{ex},0}\exp (-b\times {D}_{{ex}})={v}_{{ex}}{S}_{0}\exp (-b\times {D}_{{ex}})$$

where *S*_in,0_ and *S*_ex,0_ represent the non-diffusion weighted signals, *v*_in_ and *v*_ex_ denote the fraction of the intra- and extracellular components, ADC_r_ indicates the apparent restricted diffusion coefficient, and *D*_ex_ is the apparent *D*_ex_. The Nonlinear Least Squares method in MATLAB (version 2025a; MathWorks) with the Microstructural Analysis Toolbox for Imaging (MATI) (https://github.com/jzxu0622/mati) was used for fitting [33]. A third-party algorithm implemented in MATI was used to apply denoising as a preprocessing step [34]. Within this unified fitting framework, the *D*_in_, *D*_ex_, *f*_in_, and d were simultaneously estimated, and cellularity was calculated as *f*_in_/*d* × 100 (μm⁻¹), following previous studies [18, 19]. The parameters were constrained as follows: 0 < *d* < 50 μm, 0 < *f*_in_ < 1, 0 < *D*_in_ < 1.5 μm^2^/ms, and 0 < *D*_ex_ < 2.0 μm^2^/ms. The ADC maps were created using the assumption of mono-exponential signal decay at *b*-values of 0 and 1,500 s/mm^2^ for each diffusion time. Further, maps of relative ADC (rADC)_0Hz/40Hz_, rADC_20Hz/40Hz_, incremental area under the curve (iAUC) [35] were created through pixel-by-pixel computations using the formula as follows:

$${{{{\rm{rADC}}}}}_{0{{{\rm{Hz}}}}/40{{{\rm{Hz}}}}}=\frac{{{ADC}}_{5.8{ms}}-{{ADC}}_{51.1{ms}}}{{{ADC}}_{51.1{ms}}}\times 100$$

$${{{{\rm{rADC}}}}}_{20{{{\rm{Hz}}}}/40{{{\rm{Hz}}}}}=\frac{{{ADC}}_{5.8{ms}}-{{ADC}}_{10.2{ms}}}{{{ADC}}_{10.2{ms}}}\times 100$$

$${{{\rm{iAUC}}}}=	{\sum }_{m=2}^{{N}_{{tdiff}}}\frac{{t}_{{diff},m}-{t}_{{diff},m-1}}{2\left({t}_{{diff},{N}_{{tdiff}}}-{t}_{{diff},1}\right)}\\ 	\left[M\left({t}_{{diff},m}\right)+M \left({t}_{{diff},m-1}\right)-2M({t}_{{diff},1})\right]$$

The iAUC is a metric of signal change with respect to diffusion time and demonstrates a strong dependence on cell size [35]. T_2_ values were identified by fitting the measured signal intensities to a mono-exponential decay function.

### Statistical analysis

The Shapiro–Wilk normality test was used to evaluate data normality. Data are presented as mean ± standard deviation (SD). One-way analysis of variance (ANOVA) was conducted to assess differences in ADC values across diffusion times and microcapsule densities, as well as differences in rADC_0Hz/40Hz_, rADC_20Hz/40Hz_, and iAUC across microcapsule densities or diameters, and differences in IMPULSED-derived microstructural metrics across microcapsule densities. Post hoc multiple comparisons were conducted using Tukey’s honestly significant difference (HSD) test to incorporate multiple testing when a significant main effect was detected. Spearman’s correlation coefficient was calculated to confirm the correlation between the diameter of the microcapsule and microcapsule membrane thickness. Pearson’s correlation coefficient (*r*) was used to evaluate the correlation between diameter, *f*_in_, cellularity, and their theoretical values. Bland–Altman analysis was conducted to evaluate the reproducibility of ADC and IMPULSED-derived metrics derived from repeated imaging of phantoms fabricated in two independent preparations. GraphPad Prism version 10.6.1 (GraphPad Prism, San Diego, CA, USA) was used for all analyses. *p*-values of < 0.05 indicated statistical significance.

## Results

### Measurement of the membrane thickness of microcapsules

**Fig. 2:**
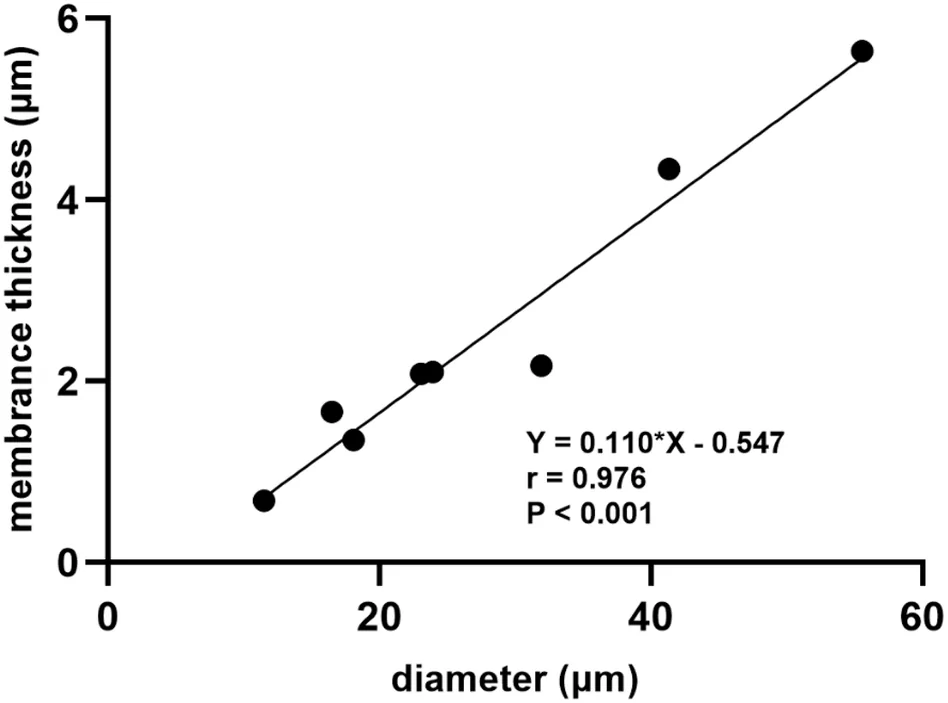
Scatter plot of microcapsule membrane thickness measurements. A strong correlation was observed between microcapsule diameter and membrane thickness (Spearman’s correlation coefficient *r* = 0.976, *p* < 0.001)

Supplemental Digital Content 3 (Figure) illustrates an example of measuring the membrane thickness of microcapsules. A strong correlation was observed between microcapsule diameter and membrane thickness (Spearman’s correlation coefficient *r* = 0.976, *p* < 0.001) (Fig. 2). Membrane thickness measurement is challenging for microcapsules with diameters of < 11.0 μm, and the membrane was assumed to be extremely thin. Based on this correlation, the association between membrane thickness and diameter was expressed as follows:

$${{{{\rm{t}}}}}_{{{{\rm{m}}}}}=0.110\times {{{\rm{d}}}}\!-\!0.547$$

where *t*_m_ denotes membrane thickness, and d represents microcapsule diameter. When correcting the diameters by subtracting the *t*_m_, the inner diameters were approximately 10.0 μm, 17.0 μm, and 25.8 μm for “small,” “medium,” and “large” microcapsules, respectively. Table 1 summarizes the theoretical volume fraction and density within the phantom calculated using the obtained inner diameters.

**Table 1:** Theoretical volume fraction and density within the phantom calculated using the obtained inner diameters

	Microcapsule								
	Small			Medium			Large		
	5.0%	10.0%	15.0%	5.0%	10.0%	15.0%	5.0%	10.0%	15.0%
Volume fraction	0.057	0.116	0.178	0.058	0.120	0.186	0.059	0.123	0.194
Density (μm^−1^)	0.570	1.160	1.771	0.342	0.706	1.097	0.223	0.478	0.753

### Temperature, T_2_ value, ADC, rADC, and iAUC

**Fig. 3:**
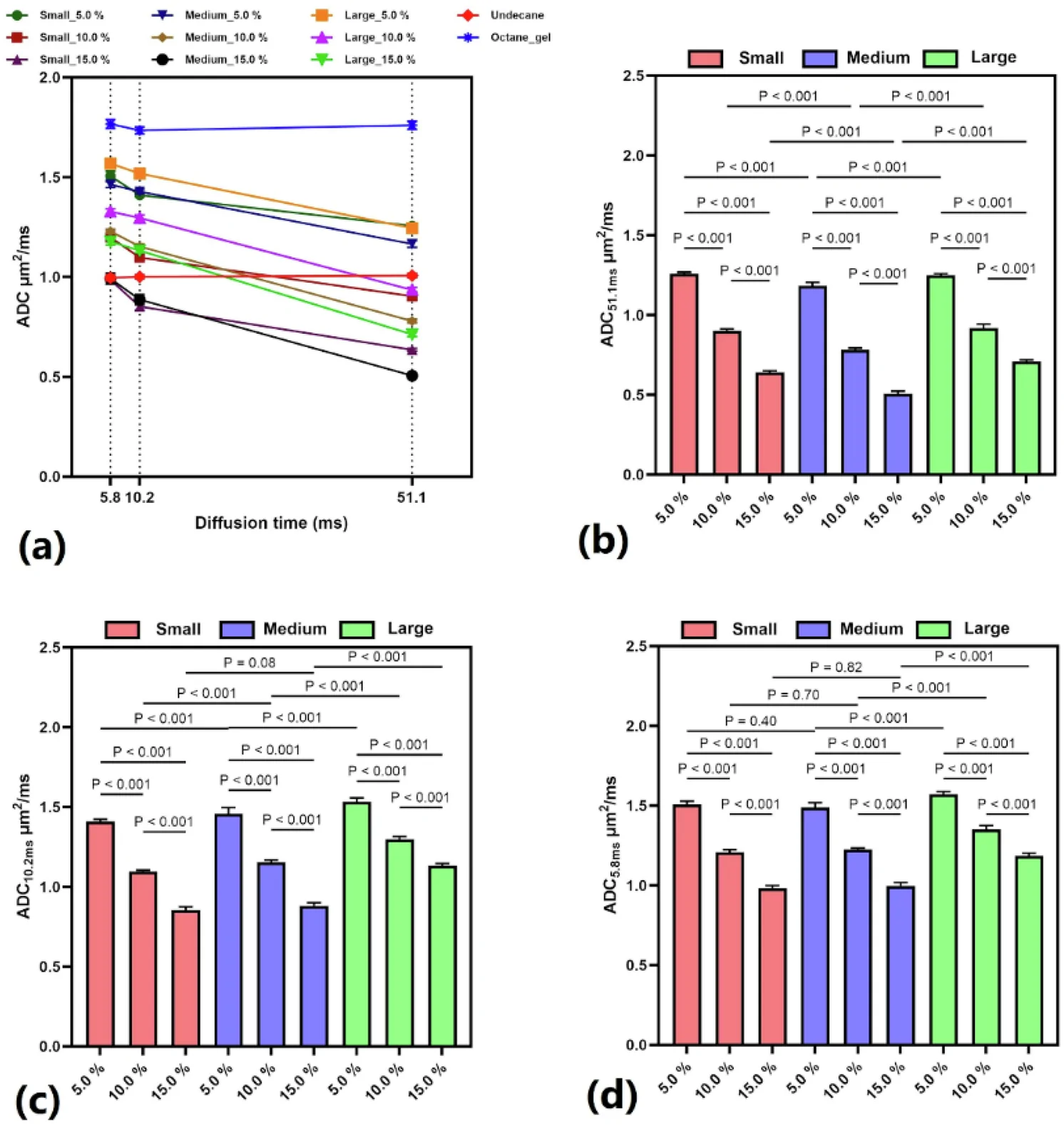
ADC values obtained from the experiments. **a** ADC values of each phantom across different diffusion times, **b** ADC values of the phantoms at diffusion times of 51.1 ms, **c** 10.2 ms, and **d** 5.8 ms. ADC, apparent diffusion coefficient; ADC_51.1ms_, ADC calculated with a diffusion time of 51.1 ms; ADC_10.2ms_, ADC calculated with a diffusion time of 10.2 ms; ADC_5.8ms_, ADC calculated with a diffusion time of 5.8 ms

**Fig. 4:**
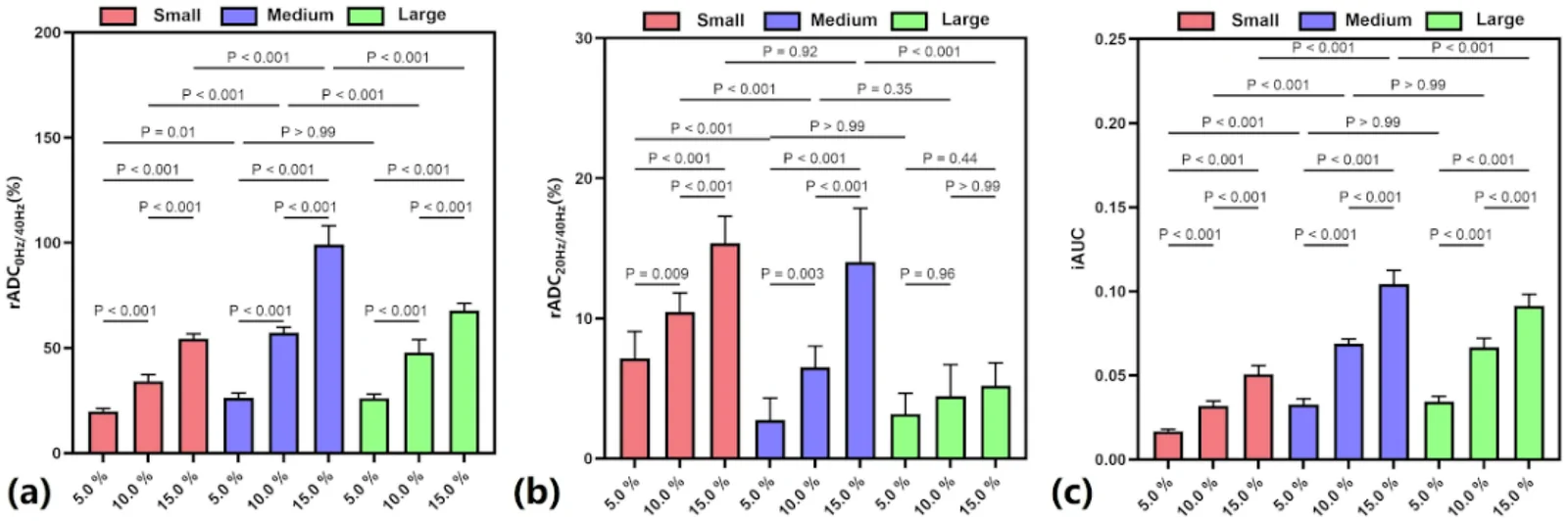
Metrics derived from time-dependent MRI. **a** rADC_0Hz/40Hz_, **b** rADC_20Hz/40Hz_, **c** iAUC. ADC, Apparent diffusion coefficient; rADC_0HZ/40Hz_, Relative change in ADC between diffusion times of 51.1 ms and 5.8 ms; rADC_20Hz/40Hz_, Relative change in ADC between diffusion times of 10.2 ms and 5.8 ms; iAUC, Incremental area under the curve

The phantom temperature between measurements was 20.7 ± 0.1 °C. The T_2_ values decreased with increasing concentration for each microcapsule diameter, with values ranging from 210 to 641 ms (Supplemental Digital Content 4 [Table]). Figures 3 and 4 and Table 2 present the PGSE- and OGSE-derived ADC, rADC_0Hz/40Hz_, rADC_20Hz/40Hz_, and iAUC values, with Supplemental Digital Content 5 (Figure) providing color maps. ADC significantly decreased with increasing diffusion time in all microcapsule phantoms (Fig. 3A, *p* < 0.001), whereas ADCs of undecane and octane gel remained constant at approximately 1.0 and 1.8 μm²/ms, respectively. In all phantoms, increasing the microcapsule density significantly decreased ADC values at all diffusion times (Fig. 3b–d, *p* < 0.001).

**Table 2:** Metrics derived from time-dependent MRI

	Microcapsule								
	Small			Medium			Large		
	5.0%	10.0%	15.0%	5.0%	10.0%	15.0%	5.0%	10.0%	15.0%
ADC_51.1ms_ μm^2^/ms	1.26 ± 0.01	0.90 ± 0.01	0.64 ± 0.01	1.18 ± 0.02	0.78 ± 0.01	0.51 ± 0.02	1.25 ± 0.01	0.92 ± 0.02	0.71 ± 0.01
ADC_10.2ms_ μm^2^/ms	1.41 ± 0.01	1.10 ± 0.01	0.86 ± 0.02	1.46 ± 0.04	1.15 ± 0.01	0.88 ± 0.02	1.53 ± 0.02	1.30 ± 0.02	1.13 ± 0.01
ADC_5.8ms_ μm^2^/ms	1.51 ± 0.02	1.21 ± 0.02	0.98 ± 0.02	1.49 ± 0.03	1.23 ± 0.01	1.00 ± 0.02	1.57 ± 0.02	1.35 ± 0.03	1.19 ± 0.02
rADC_0Hz/40Hz_ %	19.8 ± 1.4	34.4 ± 2.9	54.4 ± 2.2	26.3 ± 2.3	57.2 ± 2.6	99.2 ± 8.8	26.2 ± 1.9	47.9 ± 6.0	67.8 ± 3.4
rADC_20Hz/40Hz_ %	7.2 ± 1.9	10.5 ± 1.3	15.4 ± 1.9	2.8 ± 1.6	6.5 ± 1.5	14.0 ± 3.8	3.2 ± 1.5	4.4 ± 2.3	5.2 ± 1.6
iAUC	0.017 ± 0.001	0.032 ± 0.003	0.051 ± 0.005	0.033 ± 0.003	0.069 ± 0.003	0.104 ± 0.008	0.034 ± 0.003	0.067 ± 0.005	0.091 ± 0.007

Data is presented as mean ± standard deviation (SD)

*ADC* Apparent diffusion coefficient, *ADC*_*51.1ms*_ ADC calculated with a diffusion time of 51.1 ms, *ADC*_*10.2ms*_ ADC calculated with a diffusion time of 10.2 ms, *ADC*_*5.8ms*_ ADC calculated with a diffusion time of 5.8 ms, *rADC*_*0Hz/40Hz*_ Relative change in ADC between diffusion times of 51.1 ms and 5.8 ms, *rADC*_*20Hz/40Hz*_ Relative change in ADC between diffusion times of 10.2 ms and 5.8 ms, *iAUC* Incremental area under the curve

ADC differences relied on both microcapsule diameter and diffusion time for phantoms with nearly identical theoretical microcapsule volume fractions (Table 1). ADC_51.1ms_ and ADC_10.2 ms_ were significantly lower in the “medium” phantom than in the “small” and “large” phantoms, except at ADC_10.2 ms_ for the “small” and “medium” comparison at 3 g/20 mL (*p* = 0.08). No significant differences in ADC_5.8ms_ were found between the “small” and “medium” phantoms at any microcapsule mass, whereas ADC_5.8ms_ were consistently lower in the “medium” phantom than in the “large” phantom (Fig. 3b–d).

The rADC_0Hz/40Hz_ significantly rose with increasing microcapsule density in all phantoms (Fig. 4a). For phantoms with identical microcapsule masses, rADC_0Hz/40Hz_ was significantly higher in the “medium” phantom than in both the “small” and “large” phantoms (*p* < 0.001), except for the comparison between the “medium” and “large” phantoms at 5.0%, where no significant difference was revealed.

The rADC_20Hz/40Hz_ significantly rose with increasing microcapsule density in “small” and “medium” phantoms (*p* < 0.001), whereas no significant difference in rADC_20Hz/40Hz_ was found in the “large” phantom (Fig. 4b). For phantoms with identical microcapsule masses, rADC_20Hz/40Hz_ was significantly higher in the “small” phantom than in the “medium” phantom at 5.0 and 10.0% (*p* < 0.001). Conversely, rADC_20Hz/40Hz_ was significantly higher in the “medium” phantom than in the “large” phantom at 15.0% (*p* < 0.001), whereas no significant differences were found at 5.0 and 10.0% (*p* > 0.99 and *p* = 0.35, respectively).

The iAUC significantly rose with increasing microcapsule density in all phantoms (Fig. 4c). The iAUC for phantoms with identical microcapsule masses was significantly higher in the “medium” phantom than in the “small” phantom at all microcapsule masses (5.0, 10.0, and 15.0%; all *p* < 0.001). Conversely, the iAUC was significantly higher in the “medium” phantom than in the “large” phantom at 15.0% (*p* < 0.001), whereas no significant differences were observed at 5.0 or 10.0% (*p* > 0.99).

### Microstructural metrics

**Fig. 5:**
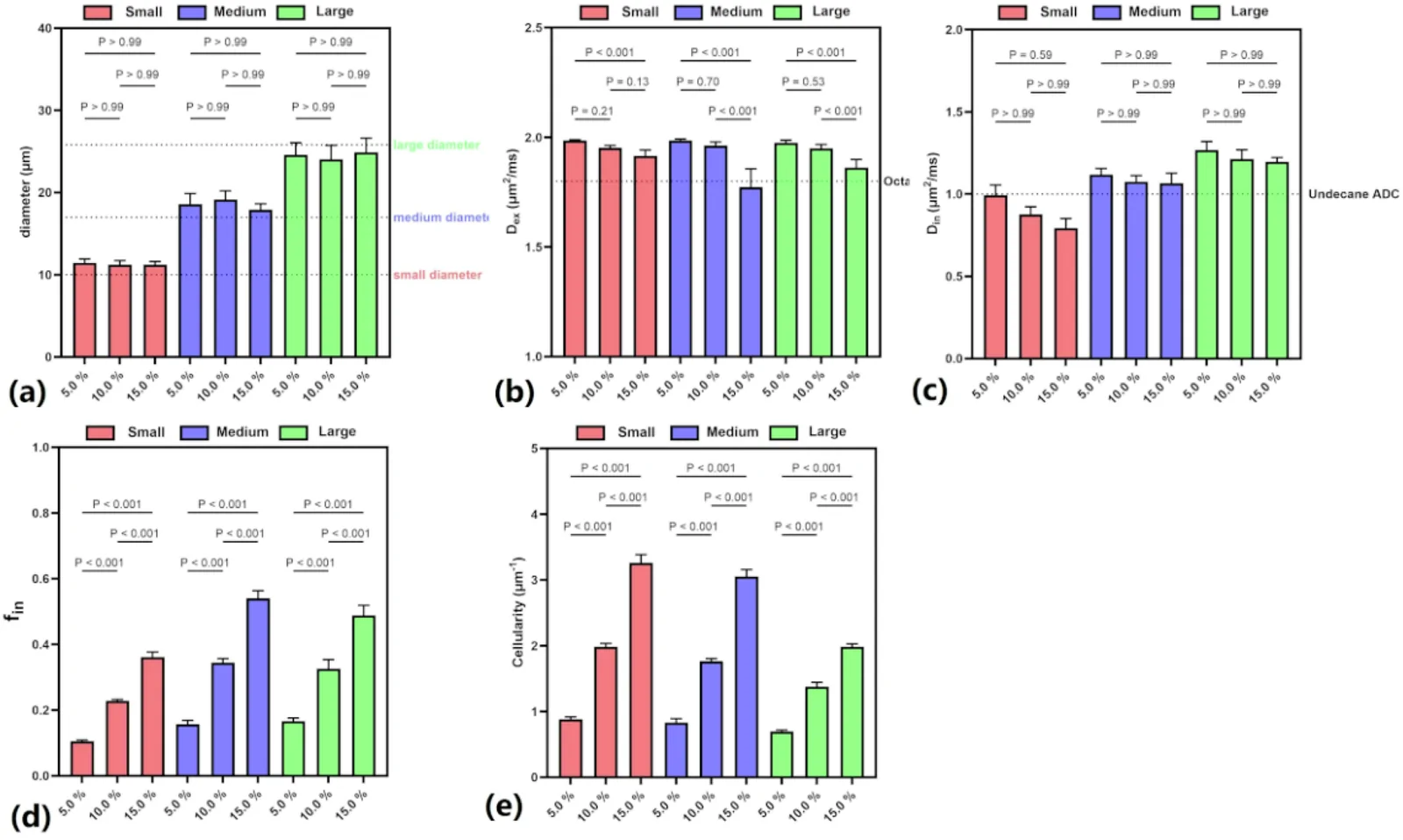
IMPULSED-derived microstructural parameters. **a** Diameter, (**b**) *D*_ex_, (**c**) *D*_in_, (**d**) *f*_in_, and (**e**) cellularity. IMPULSED, Imaging microstructural parameters using limited spectrally edited diffusion; *D*_ex_, Hindered extracellular diffusivity; *D*_in_, Intracellular intrinsic diffusivity; *f*_in_, Fraction of the intracellular component

**Fig. 6:**
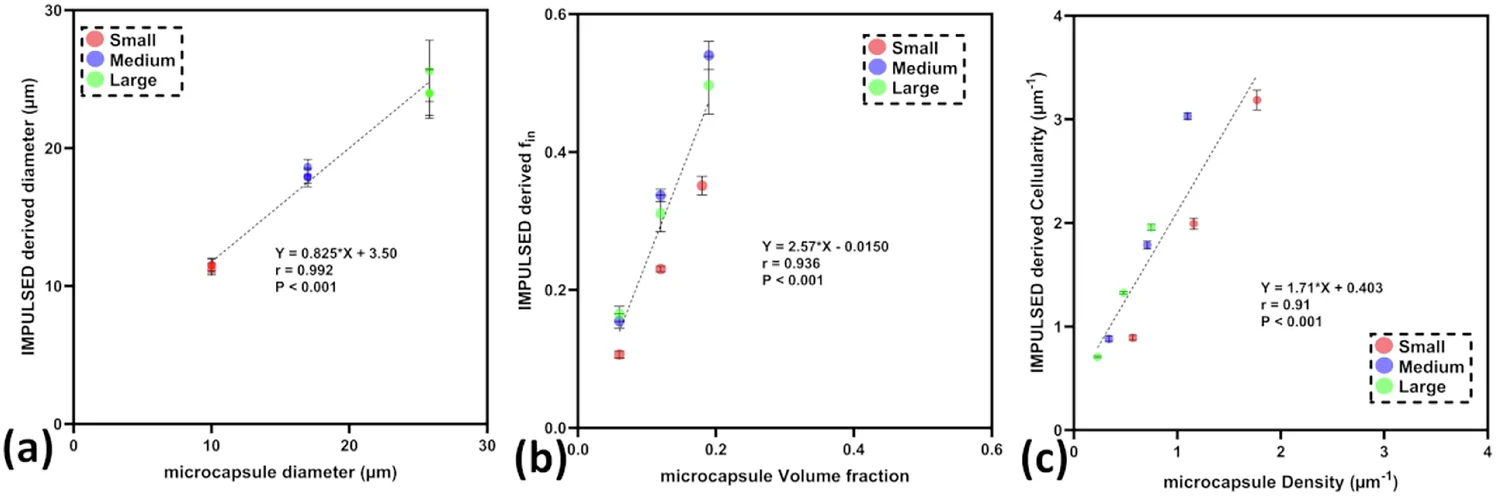
Results of correlation analyses between the theoretical values of (**a**) microcapsule diameter, (**b**) *f*_in_, and (**c**) cellularity with the corresponding values obtained from IMPULSED analysis. IMPULSED, Imaging microstructural parameters using limited spectrally edited diffusion; *f*_in_, Fraction of the intracellular component

Figure 5 and Table 3 illustrate the values of IMPULSED-derived microstructural metrics, with the corresponding color maps provided in Supplemental Digital Content 6 (Figure). IMPULSED-derived diameters were almost identical to the theoretical diameters across all microcapsule densities (Fig. 5a). As the microcapsule concentration increased from 5.0 to 15.0%, *D*_ex_ significantly decreased in all phantoms (*p* < 0.001, Fig. 5b). In the “small,” “medium,” and “large” phantoms, no significant differences in *D*_in_ were observed with changes in microcapsule concentration; however, *D*_in_ was slightly lower in the “small” phantoms and slightly higher in the “large” phantoms compared with the diffusion coefficient within the microcapsules (*i.e*., the ADC of undecane) (Fig. 5c). As the microcapsule density increased, the *f*_in_ and cellularity significantly increased for all phantoms (*p* < 0.001, Fig. 5d, e). Figure 6 illustrates correlation analyses between the theoretical values of microcapsule diameter, *f*_in_, and cellularity with the corresponding values obtained from IMPULSED analysis. The Pearson correlation coefficients revealed r = 0.992 (*p* < 0.001) for diameter (Fig. 6a), *r* = 0.936 (*p* < 0.001) for *f*_in_ (Fig. 6b), and *r* = 0.910 (*p* < 0.001) for cellularity (Fig. 6c), indicating strong correlations for all parameters. Both parameters were consistently overestimated across all phantoms, with the largest deviations observed in the “medium” phantom, although *f*_in_ and cellularity demonstrated strong correlations with their theoretical values.

**Table 3:** Microstructural metrics derived from IMPULSED analysis

	Microcapsule								
	Small			Medium			Large		
	5.0%	10.0%	15.0%	5.0%	10.0%	15.0%	5.0%	10.0%	15.0%
Diameter μm	11.5 ± 0.5	11.2 ± 0.5	11.2 ± 0.4	18.6 ± 1.3	19.2 ± 1.0	17.9 ± 0.7	24.6 ± 1.5	24.1 ± 1.7	24.9 ± 1.7
*D*_ex_ μm^2^/ms	1.99 ± 0.01	1.95 ± 0.01	1.92 ± 0.03	1.99 ± 0.01	1.96 ± 0.02	1.77 ± 0.08	1.98 ± 0.01	1.95 ± 0.02	1.86 ± 0.04
*D*_in_ μm^2^/ms	0.99 ± 0.06	0.88 ± 0.05	0.80 ± 0.06	1.12 ± 0.04	1.08 ± 0.04	1.07 ± 0.06	1.27 ± 0.05	1.21 ± 0.06	1.20 ± 0.03
*f*_in_	0.105 ± 0.004	0.228 ± 0.004	0.362 ± 0.015	0.157 ± 0.011	0.344 ± 0.013	0.541 ± 0.023	0.166 ± 0.009	0.326 ± 0.027	0.489 ± 0.030
Cellularity μm^-1^	0.88 ± 0.04	1.98 ± 0.05	3.26 ± 0.13	0.83 ± 0.06	1.77 ± 0.04	3.05 ± 0.11	0.70 ± 0.02	1.38 ± 0.07	1.98 ± 0.04

Data are presented as mean ± SD

*IMPULSED* Imaging microstructural parameters using limited spectrally edited diffusion, *D*_*ex*_ Hindered extracellular diffusivity, *D*_*in*_ Intracellular intrinsic diffusivity, *f*_*in*_ Fraction of the intracellular component

### Reproducibility

Supplemental Digital Content 7 (Figure)illustrates the Bland–Altman plots of repeated imaging of phantoms fabricated in two independent preparations. The bias for ADC was 0.647% with an SD of 2.31%, and the limits of agreement (LOA) ranged from -3.88% to 5.18% (Supplemental Digital Content 7a). The estimated microcapsule diameter demonstrated a bias of 0.944% (SD, 7.83%), with LOA from -14.41% to 16.30% (Supplemental Digital Content 7b). The bias for *D*_ex_ was -0.106% (SD, 2.18%), with LOA between -4.39% and 4.18% (Supplemental Digital Content 7c). *D*_in_ demonstrated a bias of -1.10% (SD, 7.15%), with LOA ranging from -15.12% to 12.91% (Supplemental Digital Content 7d). The bias for *f*_in_ was 1.94% (SD, 8.21%), with LOA from -14.14% to 18.03% (Supplemental Digital Content 7e), whereas cellularity exhibited a bias of -0.498% (SD, 6.62%) and LOA from -13.47% to 12.48% (Supplemental Digital Content 7f).

## Discussion

In this study, a microcapsule-based phantom was developed and evaluated. The observed time-dependent diffusion metrics and IMPULSED analysis support the microstructural validity of the proposed phantom.

### Physical validity and biological relevance

To our knowledge, this is the first diffusion phantom designed to model both intra- and extracellular water diffusion. In the present phantom, the intrinsic diffusivities inside and outside the microcapsules were similar to those commonly used in simulation studies (*D*_in_ = 1.0, *D*_ex_ = 2.0 μm²/ms) [21, 36] and reported values in tumor models: 1–2 μm²/ms for intracellular [17, 37] and 2.0–2.5 μm^2^/ms for extracellular components [19, 37, 38].

Across all phantoms, ADC significantly decreased with increasing diffusion time, reflecting improved interactions between water molecules and restricting microcapsule boundaries at longer diffusion times. Conversely, ADCs of undecane and octane gel remained constant, thereby confirming that the observed diffusion-time dependence originates from structural restriction rather than intrinsic diffusivity changes.

ADC values decreased with increasing microcapsule density at all diffusion times, consistent with clinical observations that elevated cellularity leads to reduced ADC [39, 40]. This density dependence was consistently demonstrated across all microcapsule diameters, indicating that microcapsule concentration is a dominant determinant of ADC reduction in this phantom.

The comparison of phantoms with identical theoretical volume fractions revealed that the “medium” phantom demonstrated lower ADC values than the “small” and “large” phantoms, particularly at longer diffusion times. ADC is expected to increase with increasing structure diameter under ideal conditions [41]; however, fabrication-related solid residues originating from the microcapsule shell were occasionally observed, particularly in the medium-sized microcapsules. These residues likely introduce additional structural complexity in the extra-microcapsular space, acting as diffusion barriers that trap water molecules and enhance diffusion-time dependence beyond the ideal two-compartment assumption.

Both rADC and iAUC increased with microcapsule concentration, indicating enhanced diffusion restriction at higher densities. Under identical volume fractions, rADC_20Hz/40Hz_ was highest in the “small” phantom, consistent with restricted diffusion in smaller structures. This metric is sensitive to size-dependent effects when the three-dimensional root mean square displacement (RMSD) [42] of water molecules —calculated as 5.9, 7.8, and 17.5 μm for diffusion times of 5.8, 10.2, and 51.1 ms (assuming a diffusion coefficient of 1.0 μm²/ms), respectively—is comparable to or exceeds the capsule radius. Conversely, the “large” phantom showed lower rADC_20Hz/40Hz_ as its radius substantially exceeded these RMSDs.

### Evaluation of IMPULSED analysis

The microcapsular diameters of our phantom estimated through IMPULSED analysis closely aligned with the theoretical values across all density conditions. This is consistent with previous *in vivo* studies that demonstrated good agreement between IMPULSED-derived cell diameter estimates and histopathological reference measurements [19, 23, 43].

*D*_ex_ significantly decreased with increasing microcapsule concentration. *D*_ex_ represents the ADC of the extra microcapsule space; thus, a decrease with increasing microcapsule concentration is expected. However, *D*_ex_ values were consistently overestimated relative to the ADC of octane gel (1.8 μm²/ms). This mismatch may be explained by the residual melamine residues, which hinder extra-microcapsular water; within the IMPULSED framework, such signals are mathematically misattributed to the intracellular component rather than the extracellular component.

*D*_in_ was overestimated in the “large” phantom and slightly underestimated in the “small” phantom. The intrinsic diffusivity inside the microcapsules is known in this phantom; thus, these deviations are unlikely to originate from uncertainty in the reference values. Rather, they may reflect challenges in separating intra- and extracellular signal components under specific diffusion-time and size conditions. The RMSD of water molecules may be small relative to the microcapsule diameter in the “large” phantom at shorter diffusion times, making it challenging to clearly distinguish intra- and extracellular compartments and leading to partial signal mixing and an overestimation of *D*_in_. Conversely, in the “small” phantom, even at the shortest diffusion times used in this study, the RMSD becomes larger than the microcapsule radius. Hence, the measured signal increasingly reflects restricted diffusion effects, which bias the estimated *D*_in_ toward lower values. These results may indicate that a more accurate estimation of *D*_in_ requires shorter diffusion times than those utilized in our study.

Both *f*_in_ and cellularity significantly rose with increasing microcapsule concentration and demonstrated strong correlations with their theoretical values, in agreement with previous *in vivo* studies [18, 23, 30]. However, both parameters were consistently overestimated, most pronounced in the “medium” phantom. This overestimation results from signal contamination: water molecules restricted by residues or thick membranes mimic the restricted diffusion of the intracapsular space. Within the two-compartment IMPULSED model, these signals are misassigned to the intracellular component, thereby inflating the estimated *f*_in_ and cellularity.

### Reproducibility

Bland–Altman analysis showed low bias and narrow LOA for ADC, suggesting high reproducibility in phantom fabrication. However, for IMPULSED-derived parameters other than *D*_ex_, the LOA were relatively wide despite the low mean biases. This increased variance likely reflects the inherent instability of the multi-parameter non-linear fitting process, rather than inconsistencies in the fabrication process itself.

### Limitations

This study has several limitations. First, microcapsule membranes are micrometer-scale, significantly thicker than the nanoscale membranes of biological cells [44]. Although we accounted for this by using internal diameters for theoretical calculations, these relatively thick, non-signal-producing shells reduce the available signal-contributing volume. Furthermore, the membrane thickness varied with capsule diameter [45], and for microcapsules with diameters less than 11.0 μm, the membrane was too thin to be accurately resolved by optical microscopy and was assumed to be negligible for internal diameter calculations. This may have introduced error in the reference internal diameter of phantoms. Second, organic solvents (undecane and octane) were used to model both intracellular and extracellular diffusion in the proposed phantom. While these solvents simulate target ADC values, they do not fully replicate the chemical and relaxation environment of biological tissues. Since the measured T2 values of our phantom components were not specifically tuned to match values of *in vivo* water molecules, this mismatch may influence the signal-to-noise ratio and the weighting of the diffusion signal depending on the echo time used. Future developments integrating water-based components with adjustable relaxation properties are required to achieve a more comprehensive biomimetic phantom. Third, the microcapsules used in this study demonstrated a distribution in diameter, and contamination by residual melamine membrane material occurred during the fabrication process. Recent technological advances have facilitated the fabrication of microcapsules with uniform diameters and encapsulated water-soluble substances [46]. Once this technology is fully developed, the above issues are expected to be resolved. Fourth, the microcapsules used in this study were impermeable to water, making them well-suited for the IMPULSED model. However, biological cell membranes are permeable. More advanced models, including the JOINT and exchange models [47], which extend the IMPULSED framework, enable the estimation of membrane exchange rates. The evaluation of these models requires phantoms with known membrane exchange properties. Giant liposomes are considered a promising candidate for such phantoms.

## Conclusion

In conclusion, we developed a microcapsule-based phantom designed to replicate intra- and extracellular diffusion restriction. The overall results support the validity and reproducibility of the proposed phantom. This microcapsule-based phantom provides a robust experimental platform for validating time-dependent diffusion MRI metrics and microstructural models, including IMPULSED.

## Supplementary information

**Additional file 1: Supplemental Digital Content 1 (CSV).** The distribution ratio for a given microcapsule diameter (excel tables). **Supplemental Digital Content 2 (Figure).** The pulse sequences used for IMPULSED data acquisition. OGSE acquisitions with two different frequencies (20 Hz and 40 Hz) were employed, in addition to the PGSE acquisition. **Supplemental Digital Content 3 (Figure).** An example of measuring the membrane thickness of microcapsules. The microcapsule membrane thickness for this measurement example was measured at 4.64 μm. **Supplementary Table S4.** T2 values of the phantoms used in this study. **Supplemental Digital Content 5 (Figure).** Color maps of quantitative parameters obtained from time-dependent MRI. **Supplemental Digital Content 6 (Figure).** Color maps of IMPULSED-derived microstructural parameters. **Supple. ment Digital Content 7.** Bland–Altman plots of repeated imaging of phantoms fabricated in two independent preparations. (a) ADC, (b) diameter, (c) Dex, (d) Din, (e) fin, and (f) cellularity.

## Data Availability

The datasets used and/or analyzed during the current study are available from the corresponding author on reasonable request.

## Abbreviations

ADC: Apparent diffusion coefficient
DWI: Diffusion-weighted imaging
IMPULSED: Imaging microstructural parameters using a limited spectrally edited diffusion
OGSE: Oscillating gradient spin-echo
PGSE: Pulsed gradient spin-echo
